# Monolithic optical resonator for ultrastable laser and photonic millimeter-wave synthesis

**DOI:** 10.1038/s42005-024-01660-3

**Published:** 2024-06-04

**Authors:** Wei Zhang, Eric Kittlaus, Anatoliy Savchenkov, Vladimir Iltchenko, Lin Yi, Scott B. Papp, Andrey Matsko

**Affiliations:** 1 Jet Propulsion Laboratory California Institute of Technology, 4800 Oak Grove Drive, Pasadena, CA 91109-8099 USA; 2 National Institute of Standards and Technology, 325 Broadway, Boulder, CO 80305 USA

**Keywords:** Optical metrology, Microwave photonics

## Abstract

Optical resonators are indispensable tools in optical metrology that usually benefit from an evacuated and highly-isolated environment to achieve peak performance. Even in the more sophisticated design of Fabry-Perot (FP) cavities, the material choice limits the achievable quality factors. For this reason, monolithic resonators are emerging as promising alternative to traditional designs, but their design is still at preliminary stage and far from being optimized. Here, we demonstrate a monolithic FP resonator with 4.5 cm^3^ volume and 2 × 10^5^ finesse. In the ambient environment, we achieve 18 Hz integrated laser linewidth and 7 × 10^−14^ frequency stability measured from 0.08 s to 0.3 s averaging time, the highest spectral purity and stability demonstrated to date in the context of monolithic reference resonators. By locking two separate lasers to distinct modes of the same resonator, a 96 GHz microwave signals is generated with phase noise -100 dBc/Hz at 10 kHz frequency offset, achieving orders of magnitude improvement in the approach of photonic heterodyne synthesis. The compact monolithic FP resonator is promising for applications in spectrally-pure, high-frequency microwave photonic references as well as optical clocks and other metrological devices. ©2024. All rights reserved.

## Abstract (web summary)

Optical resonators are essential tools for high precision metrology and applications where the spectral purity is highly demanded. Here, the authors demonstrate a monolithic resonator made of fused silica to support 18 Hz integrated laser linewidth in the ambient environment, and W-band microwave generation with low phase noise of -100 dBc/Hz at 10 kHz frequency offset.

## Introduction

Ultrastable lasers are critical for advanced research and applications requiring high-spectral purity, such as optical atomic clocks^1^, photonic microwave generation^2,3^, communications^4^, and very long baseline interferometry^5^. The combination of Fabry–Perot (FP) cavities as frequency references and the Pound–Drever–Hall (PDH)^6^ locking technique represents the “golden rule” for laser frequency stabilization, leading to state-of-the-art frequency stability on the level of 10^−16^ or better^7–11^. To support such quiet operation, a sub-femtometer level of cavity length fluctuation is required. This high demand typically necessitates that the FP cavity should be operated in a laboratory environment with a complex isolating system comprising of the maintained vacuum, temperature stabilization (either at room temperature or cryogenics), and vibration isolation, leading to relatively large size, weight, and the power consumption (SWaP).

A growing research activity seeking to demonstrate high-stability lasers with minimization of SWaP arises from potential fieldable applications, such as geodesy^12^, space-based operation^13,14^, or transportable optical clocks^15^, where system robustness, as well as integration, are primary considerations. Besides efforts centered around the sophisticated design of the FP cavity and its support structures^16,17^, monolithic reference resonators provide an alternative solution. In this case, the optical field is mostly confined to the host material of the solid-state resonator, so the physical parameters of this medium are directly related to the achievable performance. For instance, material attenuation limits the quality factor of a resonator and, thus, the phase noise and stability of the device. The environmental sensitivity of the material readily imprints on the environmental sensitivity of the resonator. The nonlinearity of the material restricts the maximum intracavity power and hence limits the achievable signal-to-noise ratio of the system, restricting both short-term stability as well as the spectral purity of an oscillator. With careful design, several types of monolithic resonators to date have been successfully developed to reach 100 Hz linewidth level or less, including chip-scale waveguides^18,19^, whispering-gallery-mode resonators (WGMR)^20–24^, and microrods^25^. In contrast to microfabricated devices, monolithic photonic resonators based a bulk-optic and cylindrical cavity have demonstrated the narrowest linewidth among dielectric reference cavities^26^, thanks to the combination large mode volume, design flexibility, and narrow linewidth of the resonator modes.

In this article, we report an advanced monolithic photonic resonator for laser stabilization that achieves thermal-noise-limited operation in the ambient environment. Optimization of cavity fabrication by using low-loss fused silica (Suprasil 3001) permits resonator finesse of 170,000. By engineering the cavity geometry and support structure, technical noise is suppressed so that the phase noise of the resonator-stabilized laser is limited only by thermorefractive noise^27,28^ at the close-to-carrier frequency, and achieves −112 dBc/Hz at 10 kHz. The corresponding integrated linewidth is 18 Hz, among the narrowest results reported to date for monolithic resonators. The fractional frequency stability is 7 × 10^−14^ after subtracting a constant 160 Hz/s linear frequency drift. This performance level is attained with the resonator installed in a compact enclosure of 0.23 L volume, without vacuum or additional vibration isolation.

Beyond applications in spectrally pure stable lasers, the monolithic resonator permits a simple approach to low-noise photonic millimeter-wave synthesis^22^. As a comparison, the conventional electronic oscillators degrade the phase noise at higher frequencies generated through multiplier chains, regardless of 10–100 W power consumption. The frequency combs either based on the modelocked laser^2^ or microresonator^29^ allow low noise optical-to-microwave synthesis in lab environment, however the complexity of the system constrains their fieldable applications. In this article, we separately lock two lasers to distinct modes of the photonic resonator and down-mix their heterodyne-beatnote from optical to microwave frequencies by using a fast photodiode, translating the superb low-phase noise of the resonator to the radiofrequency domain. In this photonic synthesis approach, the phase noise of the heterodyne signal is independent of the output frequency. We demonstrate 4 and 96 GHz signals with single sideband phase noise of −100 dBc/Hz at 10 kHz, representing a simple approach for low-noise millimeter-wave synthesis via optical-to-microwave conversion. Through these experiments, the maximum frequency was limited only by the photodiode and characterization apparatus available; looking forward, the same performance level can be translated to sub-millimeter wave or THz frequencies by using higher frequency photomixers^30,31^, offering new capabilities for coherent THz sensing and high-frequency radars. Altogether, these results establish an alternative promising approach to achieve high-performance reference cavities and photonic mm-wave synthesis while permitting ambient environment operation in a robust, compact system.

## Results

### Optical frequency noise and stability

**Fig. 1:**
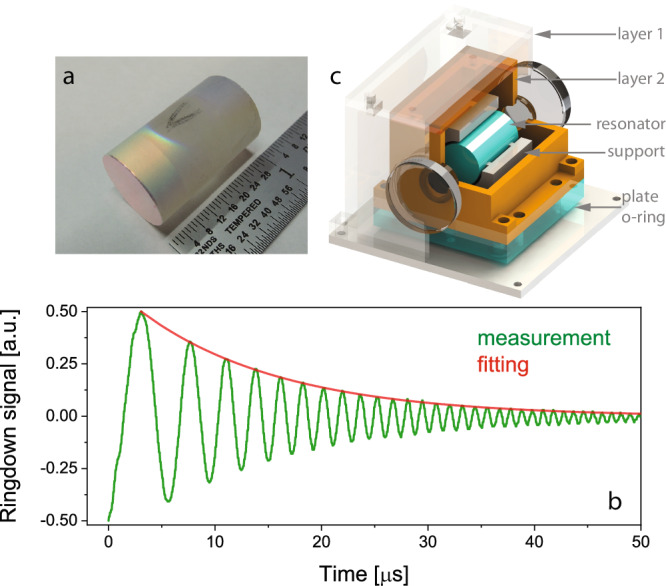
Photonic resonator and the assembly. **a** Photograph of the photonic resonator. **b** The cavity ring-down (green) in reflected field when the laser frequency is swept crossing the resonance and the exponential decay fitting (red) with 13.3 μs time constant leading a finesse of 170,000. **c** The section view of cavity assembly, including the two-layer thermal shield and the resonator. The heater and thermistor are attached to layer 1 for temperature stabilization.

The photonic resonator is fabricated from a single piece of high-purity fused silica glass. As shown in Fig. 1a, the length of the resonator is 25.4 mm, and the diameter is 15 mm, corresponding to a total volume of about 4.5 cm^3^. Both facets of the resonator, which is plano-convex with a 1 m radius of curvature (ROC), are super-polished to reduce scattering. To establish the resonance, high reflective dielectric layers centered at 1550 nm are deposited on both facets of the resonator. The transmission of the coating is designed to be 10 part-per-million (ppm), and the loss is <1 ppm. Figure 1b shows the measurement of the optical field ringdown (green) in reflection, and the fitting curve (red) reveals a 13.3 μs decay constant, yielding a finesse of 170,000. Combined with the measurement of the intensity of the reflection and transmission, the cavity transmission and loss (mostly from material absorption and scattering) are verified to be 7.9 and 10.5 ppm^32^, respectively, which are consistent with the expected values. The corresponding cavity linewidth is 24 kHz, supporting a quality factor of about 8.3 billion at this wavelength. The birefringence of the cavity leads to two polarization states with a frequency separation of about 30 MHz, which has a negligible impact on laser frequency stabilization by launching a beam with circular polarization for PDH locking^6^.

To perform as a thermal-noise-limited frequency reference in the ambient environment, the vibration- and temperature-induced frequency fluctuations are two critical technical noise sources that must be significantly suppressed. With no additional vibration isolation, the vibration sensitivity of the resonator is minimized by previously reported strategies^26^ to achieve 10^−10^/*g* along the gravitational direction and horizontal plane. As shown in Fig. 1c, the cavity is installed in a support structure that is 25.4 mm in length, made from Teflon, and symmetrically held by four supporting teeth. According to finite element analysis, the distance between the supporting teeth and the facet of the cavity and the open angle of the tooth are chosen to be 4.35 mm and 60°, respectively. The impact of environmental temperature fluctuations on the resonator is suppressed by thermal isolation consisting of two layers and cavity support. Figure 1c shows a cross-sectional view of the enclosure. The temperature fluctuation on layer 1 (copper, 60 mm × 70 mm × 55 mm) is stabilized to mK level by using a resistive heater. Layer 2 (copper) is bolted down to layer 1 through a plate (Teflon) with three o-rings (Viton) on the bottom to reduce the thermal conductivity. The cavity support (Teflon) is installed in layer 2, and thermal damping is provided by the material and the minimization of the contact area with the resonator. The thermal isolation is equal to a second order resistor–capacitor low pass filter, and the temperature-induced frequency fluctuation is 8.5 × 10^−13^*τ*, where *τ* is the averaging time.

**Fig. 2:**
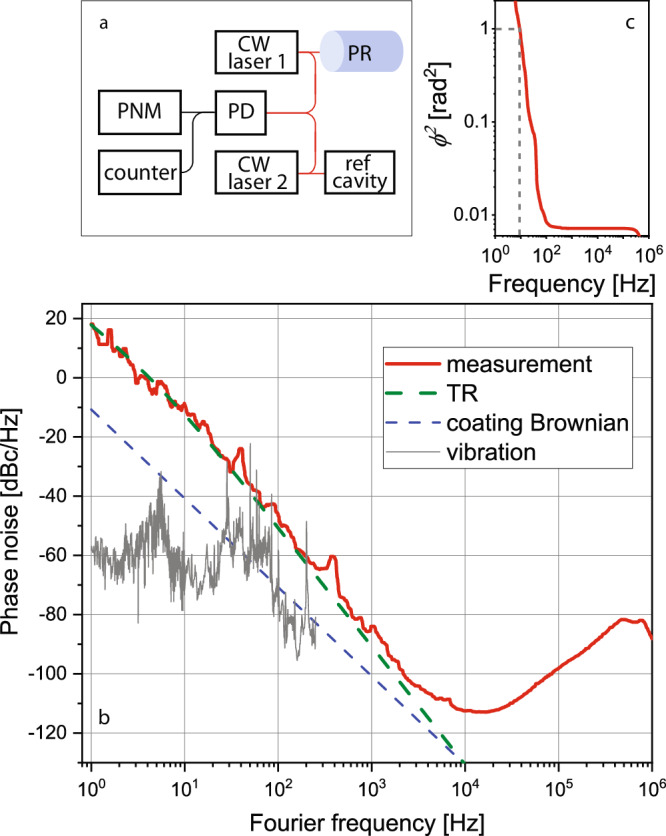
Laser frequency stabilization based on the photonic resonator. **a** Schematic of frequency stabilization and phase noise measurement. CW laser continuous-wave laser, PR photonic resonator, PNM phase noise measurement, PD photodetector. **b** Phase noise measurement (red) representing the stability of resonator-stabilized laser in the frequency domain. Thermorefractive noise (TR, green-dashed) sets the noise floor up to 1 kHz, and the thermal Brownian noise (blue-dashed) and derived vibration-induced noise are well below the measurement. **c** The integrated phase noise. The phase error of 1 rad^2^ corresponds to 9 Hz, leading to the FWHM laser linewidth of 18 Hz.

Figure 2a presents the scheme of the laser frequency stabilization on the photonic resonator and the characterization of the stability. A continuous-wave laser (CW laser 1) at 1550 nm is frequency-locked on the photonic resonator and heterodyned with the reference laser (CW laser 2). The beatnote signal is recorded by a phase noise analyzer and a frequency counter for analysis. The measured noise performance is almost exclusively attributed to the photonic resonator since the contribution from the reference laser is negligible (see Methods). Figure 2b shows the power spectral density (PSD) of the phase noise measurement (red line). For Fourier frequencies from 1 Hz to 3 kHz, the laser noise is limited by thermorefractive noise (TR, green-dashed line)^27,28^ arising from the monolithic fused silica spacer. Around 10 kHz, the laser noise is at −112 dBc/Hz, which is limited by the PDH detection noise, including shot noise and detector impedance noise. For frequencies above 20 kHz, the servo inloop errors from each of the two stabilized lasers are the dominant contribution; the servo bump at 750 kHz is from the reference laser, and the 500 kHz feature is from the resonator-stabilized laser. The vibration-induced noise (gray line) is derived by multiplying the vibration noise on the breadboard where the resonator is sitting, and the simulated vibration sensitivity is well below the TR noise floor. The laser linewidth is determined by integrating the PSD of the phase noise from high Fourier frequencies towards zero^33^. Figure 2c shows the full-width half-maximum (FWHM) laser linewidth corresponding to the integrated phase noise up to 1 rad^2^ is 18 Hz, dominated by the TR-noise limit at 15 Hz.

**Fig. 3:**
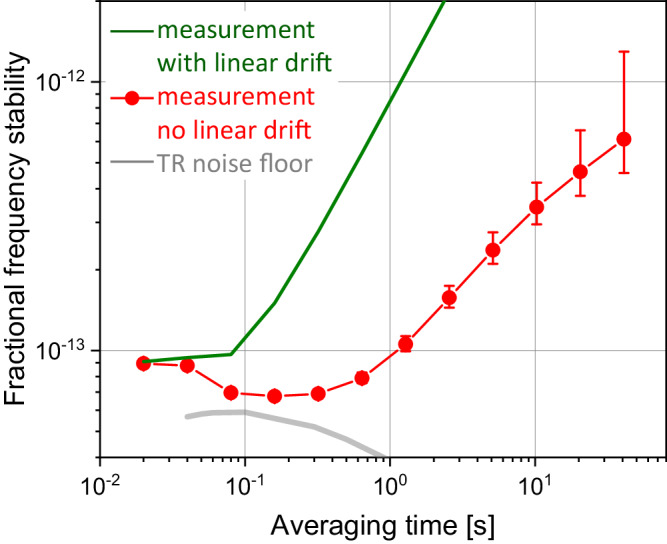
Fractional-frequency stability (FFS) measurement in time domain. Green: FFS with linear drift. Red-circle: FFS with linear drift removed, achieving 7 × 10^−14^. The error bar accordingly shows 1σ confidence interval.  Gray; predicted thermorefractive (TR) noise.

Figure 3 presents the fractional frequency stability (FFS) of the resonator-stabilized laser for time domain analysis. The beatnote signal is recorded by a *λ*-type, dead-time-free frequency counter to compute the Allan deviation. With the linear drift (160 Hz/s), the FFS (green line) reaches <1 × 10^−13^ for *τ* < 0.08 s, and 8.5 × 10^−13^*τ* due to linear drift for *τ* > 0.1 s. After correcting for the linear drift in data processing, the FFS (circle-line, with error bar representing 1σ confidence interval) achieves 7 × 10^−14^ for 0.08 s < *τ* < 0.3 s, approaching the TR-noise-limit (gray) at 5 × 10^−14^ and dominated by residual nonlinear frequency drift for *τ* > 1 s.

### Photonic generation of W-band signals

As an example application of the superb laser noise enabled by the photonic resonator, we next use it to construct a millimeter-wave oscillator based on the heterodyning of two lasers locked to distinct cavity modes. Typically, heterodyne down-mixing of off-the-shelf lasers will result in an output RF signal with relatively high phase noise given by the quadrature sum of the phase noises of the two independent lasers. The ultra-stable photonic resonator offers two benefits. First, locking a laser to the resonator greatly reduces phase noise and improves long-term frequency stability via active feedback. Second, two independent lasers can be separately locked to disparate modes in the same resonator, permitting common-mode rejection of technical noise related to the resonator environment.

**Fig. 4:**
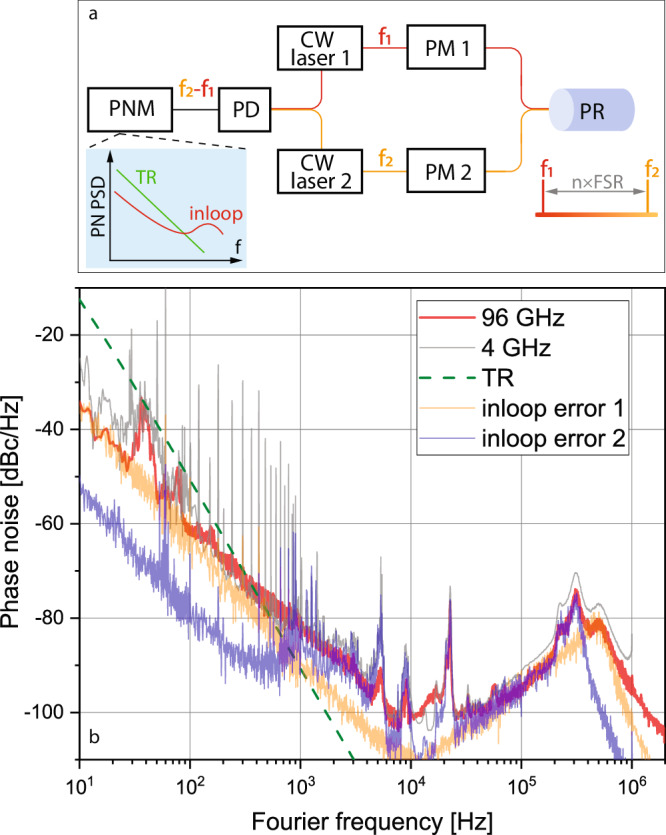
The W-band microwave generation based on the photonic resonator and phase noise measurement. **a** Schematic. CW laser continuous-wave laser, PM phase modulation, PNM phase noise measurement. The two lasers with center frequency at f1 and f2, respectively, are locked to separated photonic resonator (PR) modes simultaneously with frequency difference at a multiple of free-spectral range (FSR). Inset: The expected phase noise of the heterodyned signal demodulated by the photodetector (PD) is below the thermorefractive (TR) noise of individual laser at low Fourier frequencies, and limited by inloop error of the PDH locking. Phase noise measurement. **b** Phase noise measurement. Red: phase noise at carrier frequency of 96 GHz. Orange and blue: PDH inloop error for laser 1 and 2, respectively. Gray: phase noise at a carrier frequency of 4 GHz. Green dash: thermorefractive noise.

To evaluate the utility of the stable photonic resonator to millimeter-wave signal generation, we implemented a dual stabilized laser system as diagrammed in Fig. 4a. Two CW lasers are operating around two distinct cavity modes, and the corresponding beatnote signal at *f* = 96 GHz is detected (see Methods). The inset of Fig. 4a illustrates the expected phase noise of the millimeter-wave signal based on the dual stabilized laser system. One laser locked to one resonator mode should experience a nearly identical (coherent) thermal fluctuation process (dominated by TR noise) as the other laser on a distant mode. The incoherence between the two lasers is contributed by the difference in beam size, which is negligible in 100 GHz space. As a result, the phase noise of the heterodyned signal shows a common noise rejection relative to the TR noise and is limited by the incoherent contribution, i.e., the residual noise of PDH locking for each laser. The noise rejection, either in amplitude or ineffective Fourier frequency where TR noise is equal to measured phase noise, is determined by the free-running noise of each laser and the capability of the frequency locking.

For direct phase noise characterization of the 96 GHz signal, we implemented a hybrid microwave-photonic frequency discriminator using a fiber optic delay line (see Methods)^22,34,35^. The measured phase noise of the output signal at a carrier frequency of 96 GHz is plotted in Fig. 4b, revealing phase noise of below −60 dBc/Hz at 100 Hz offset and below −100 dBc/Hz at 10 kHz offset. These close-in phase noise results are around 100 × lower than our prior results using lasers injection-locked to two separate microresonator cavities^22^, resulting from both the superb stability of the monolithic photonic resonator and the common-mode cancellation of technical noise achieved by locking both lasers to a single resonator. The inloop error from laser 1 limits the phase noise from DC to 1 kHz, and the servo bump due to feedback to PZT of laser 2 dominates from 1 to 10 kHz. and the combined servo bump from the two laser locking by current modulation is seen above >10 kHz, in which the 500 kHz corresponds to laser 1 and 300 kHz is from laser 2. Note the spikes around 10–20 kHz from laser 2 elevates the measured noise level, and could be mitigated by using a laser without this intrinsic noise. The output phase noise of the resonator-based source was corroborated by direct measurements with frequency detuning between the two locked lasers reduced to 4 GHz (gray), despite negligible difference around the servo bumps. In principle, this signal generation can be tuned to any multiple of the cavity FSR, provided a suitable laser is available. As long as the phase noise of the locked laser does not change, then the beat-note phase noise is essentially independent of output frequency. As a result, the same performance could be extended to the sub-mm wave or THz regime by replacing the commercial photodiodes used with a THz photomixer^30,31^.

### Discussions and conclusions

To illustrate the high spectral purity enabled by our approach in the context of state-of-the-art monolithic, vacuum-free reference resonators, Table 1 presents the comparison of the integrated laser linewidth^33^ <100 Hz and measured Allan deviation for recent systems. A laser locked to a mm-scale fused silica microrod^25^ achieves a thermal-noise-limited laser linewidth of 62 Hz. By increasing the mode volume, a laser locked to an on-chip coil^19^ can narrow the linewidth to 36 Hz. Self-injection locking a laser to the WGMR based on crystalline MgF_2_^21^ achieves similar results. A prior design for the photonic resonator with 20,000 finesse^26^, due to the material loss of the fused silica, demonstrates the linewidth of 25 Hz. In this work, there are three improvements summarized as follows to upgrade the frequency stability presented in^26^. In a general aspect, the resonator has a larger ROC on the facet (1 m vs 0.5 m), leading to thermal-noise-limit laser linewidth (15 Hz vs 21 Hz). For the improvement in frequency domain measurement, the higher finesse (170,000 vs 20,000) due to lower loss is critical. The enhanced signal-to-noise ratio of PDH locking allows a tight locking up to 300 Hz (red line in Fig. 2b). The improvement in the time domain comes from the enhanced thermal isolation, leading to a more deterministic linear drift. Though the FFS with linear drift (green line in Fig. 3) is approximately a factor of 2 improvements compared with the results in^26^, the FFS with linear drift removed can achieve <10^−13^, approaching the thermal noise floor (gray line in Fig. 3). Both measurements in frequency and time domains achieve the best performance so far among monolithic optical references.

**Table 1:** Comparison of the stability performance based on non-vacuum, monolithic optical resonators

Resonator	Laser linewidth (Hz)	Allan deviation (*τ*)
Microrod^25^	62	3 × 10^−13^ (10–100 ms)
Chip coil^19^	36	2 × 10^−13^ (1–10 ms)
WGMR^21^	30	3 × 10^−13^ (10–20 ms)
Bulk FP^26^	25	1 × 10^−13^ (20 ms)
This work	18	7 × 10^−14^ (80–300 ms)

There are several aspects that would likely improve the performance of the photonic resonator. (1) Finesse. Since the loss of the resonator is measured at ~10 ppm, the finesse can be further increased by >250,000 by reducing the transmission while keeping sufficient cavity contrast for PDH locking with low residual locking noise. To intrinsically reduce the loss of the resonator, we are investigating the material properties of fused silica and the manufacturing process. (2) Thermorefractive noise floor. The ROC of the surface can be readily increased up to 10 m, which enlarges the mode size to lower the thermorefractive noise floor. Using crystalline materials^36^ of which the thermorefractive index is lower than fused silica would be a possible solution, though other technical factors (e.g., thermal expansion and vibration sensitivity) should be considered. With these two practical improvements, the photonic resonator can support the stabilized laser with sub-10 Hz integrated linewidth, a performance typically requiring vacuum-gap FP cavities with compact size^37^. To reach similar frequency stability, a recent work^38^ presents a 20 mm FP cavity, which is assembled in a vacuum and thus does not require a continuous running vacuum pump during the operation. It is worth mentioning that the coherent cancellation of the thermal noise^39–41^ might be a path to reduce the TR noise of the photonic resonator. (3) The residual amplitude modulation (RAM)^42^ and frequency drift compensation. The laser frequency stabilization based on the monolithic resonator can afford up to 300 ppm (FFS ≈ 4 × 10^−14^), which is comparable with the resonator thermal noise floor. Though the free-running RAM is highly up to the PDH setup and the temperature control of the EOM, such an amount of RAM contribution would need a relatively long averaging time to dominate the overall stability performance. For further development of this resonator with more deterministic linear drift or with drift cancellation^36,43^ by which the FFS can be maintained on 10^−14^ level, the RAM stabilization^42,44^would be necessary.

**Fig. 5:**
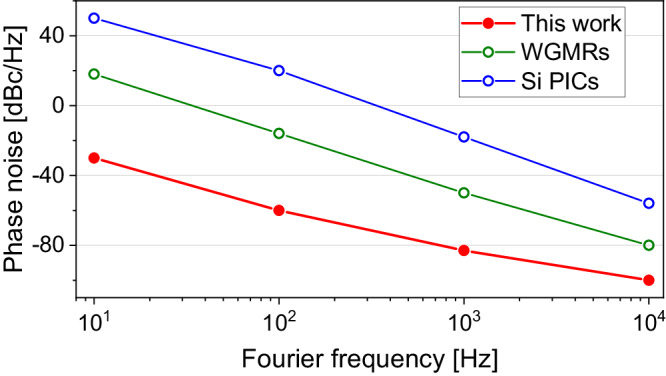
Comparison of the phase noise results based on photonic heterodyne approaches for millimeter-wave generation. Green: WGMRs^22^. Blue: Si PICs^45^. Red: duplication of the result in Fig. 4b.

Moreover, the photonic resonator enables a relatively simple and robust approach to highly coherent photonics-based millimeter-wave generation. Figure 5 presents a comparison of the phase noise in the avenue of photonic heterodyne approaches. The green line shows the results in ref. ^22^, where two semiconductor lasers are injection-locked to separated WGMRs, and the blue line presents the self-injection-locked based on Si photonic integrated circuits^45^ (Si PICs). The phase noise based on locking lasers to a common photonic resonator, as shown in Fig. 4b, exhibits up to 80 dB improvement for Fouirer frequencies from 10 Hz to 10 kHz. Since the phase noise presented in Fig. 4b is limited by the inloop error of the locking, it can be improved by using a laser with lower free-running noise. We have achieved −110 dBc/Hz at 10 kHz (10 dB improvement over Fig. 4b result) by using another RIO PLANEX as laser 2, though the frequency distinct is 4 GHz from laser 1. The low noise photonic millimeter-wave synthesis may find use for an array of sensing and communications applications. For example, low-noise oscillators operating directly at high frequencies can directly enhance the performance of future radar instruments, offering improvements to resolution^46^, reconfigurability^47,48^, and sensitivity^22,49^. Phase noise level, in particular, impacts the achievable dynamic range of pulse-compressed (such as modulated cw) radar measurements, and as a result, the low transmitted noise level will be key to suppressing clutter in future Earth-observing scientific radar instruments^50,51^.

## Methods

### Laser frequency stabilization

As shown in Fig. 2a, we perform PDH locking to stabilize the frequency of CW laser 1 at 1550 nm to the photonic resonator. Using a polarization-maintaining fiber coupler, only about 10 μW is sent to the resonator for frequency locking, while the majority of the laser power (several mW) is reserved for characterization or other applications. A fiber-based waveguide electro-optic modulator (EOM) implements phase modulation at 9.8 MHz to generate the locking error signal, while the laser current modulation port is used as the actuator for frequency lock. To investigate the frequency stability, CW laser 1 locked on the resonator is heterodyned with the reference laser, i.e., CW laser 2 locked on a 5-cm cubic vacuum-gap FP cavity fabricated from ultralow expansion glass. The frequency stability of the reference laser is 2 × 10^−15^, which is well below the TR noise of the photonic resonator. Due to the narrow cavity linewidth, the RAM-induced noise^42^ is substantially suppressed. Consequently, 1 ppm RAM corresponds to 0.024 Hz frequency fluctuation, which is a negligible level compared to the TR noise. In-line isolators are inserted into the locking scheme to prevent unexpected interference. The temperature of the EOM is controlled to stabilize the long-term RAM drift.

### W-band microwave signal generation

We implemented a dual stabilized laser system as diagrammed in Fig. 4a. Two CW lasers (RIO PLANEX labeled as laser 1 and Velocity TLB 6700 as laser 2) are operating around two distinct cavity modes, 193.4 THz (*f*_1_) and 193.496 THz (*f*_2_), respectively. Each laser has independent phase modulation (9.8 MHz for Laser 1 and 60.2 MHz for Laser 2) provided by the corresponding fiber-based EOM. In-line isolators are placed in the system to eliminate back-reflection and unexpected interference. The two lasers are combined by a fiber coupler (50/50 coupling ratio) and launched into the cavity. Approximately 2 μW optical power from each laser is received by the photodetector for PDH error signal generation, which is demodulated at the corresponding frequency. The laser 1 is frequency-stabilized by controlling the current, and laser 2 is by current and piezoelectric transducer (PZT). The majority part of the optical power from each laser, approximately 10 mW, is combined by a fiber coupler and incident on a fast photodiode (Finisar XPDV4120R, bandwidth > 90 GHz), leading to an output power of 150 μW at *f* = 96 GHz, corresponding to 24 free-spectral range (FSR) of the resonator. This is then boosted to 11 dBm (12.5 mW) using a W-band amplifier (Eravant BP-7531142515-1010-E1) for signal analysis.

### Phase noise measurement of the 96 GHz signal

The basic approach is as follows: a second copy of the W-band signal is synthesized by picking of some light from each laser using fiber-optic couplers. A frequency detuning of Δ = + 100 MHz in this signal is implemented by shifting the optical frequency in one of the arms using an acousto-optic modulator driven by a low-noise RF signal generator (Keysight E8257D). After combining these two reference optical signals into a single optical fiber, a variable fiber delay line is inserted before the signal is down-converted using another nominally identical fast photodiode. The result is two copies of the W-band signal, separated in frequency by Δ and in time by delay *τ*_*d*_. To complete the frequency discriminator approach, these two signals are mixed down to Δ using a W-band mixer (Hasco HWMX10-SFW), and the characteristic spectral shape is digitized using a radiofrequency spectrum analyzer (Keysight N9030B). To eliminate nulls in the frequency discriminator transfer function, several delay lengths (0.2, 1.0, 3, 4.3, 10.5, 11.6, and 14.8 km) are used^22^.

## Data Availability

The data that support the plots within this paper and other findings of this study are available from the corresponding author upon reasonable request.
